# Nested Continual Learning with Elastic Weight Consolidation for Progressive Alzheimer’s Disease Classification from MRI

**DOI:** 10.2174/0115734056512153260820074645

**Published:** 2026-08-31

**Authors:** Abdurrahim Akgündoğdu, Şerife Çeli̇kbaş

**Affiliations:** 1 Department of Electrical and Electronics Engineering, Istanbul University-Cerrahpaşa, İstanbul, Türkiye;; 2 Biomedical Device Technology, Namık Kemal University, Tekirdağ, Türkiye

**Keywords:** Alzheimer’s disease, Magnetic resonance imaging, Continual learning, Hierarchical classification, Catastrophic forgetting, Elastic weight consolidation, Deep learning

## Abstract

**Introduction/ Objective:**

MRI-based Alzheimer’s staging is often modeled as separate tasks, although clinical assessment progresses from screening to more refined staging. This study developed a continual-learning framework for retaining earlier diagnostic knowledge.

**Methods:**

A nested multi-head framework was implemented for sequential learning of related binary classification tasks from structural MRI slices. Because verified subject identifiers were unavailable in the Mendeley dataset, the primary experiments were kept at the level of slice-based methodological analysis. Subject-level external validation was additionally carried out on OASIS-VBM with subject-wise three-fold cross-validation.

**Results:**

In the Mendeley slice-level evaluation, the proposed EWC + replay model retained high post-sequence discrimination, with AUC values of 0.976, 0.985, and 1.000 for T1, T2, and T3. The ECE values were 0.034, 0.057, and 0.187. When the model was tested at the subject level on OASIS, AUC values decreased to 0.753, 0.748, and 0.749. This difference shows that the slice-level results should be interpreted cautiously.

**Discussion:**

The findings support the use of the proposed framework for studying sequential retention and forgetting in hierarchical AD staging. However, Mendeley results are methodological findings at the slice level and should not be interpreted as patient-level clinical performance.

**Conclusion:**

This study presents a nested continual-learning framework for hierarchical Alzheimer’s disease staging. The framework was tested with ablation, calibration, baseline, and subject-level validation analyses. The results show that it can be used to study retention across related AD staging tasks. Future studies using subject-wise longitudinal cohorts, 3D models, and multimodal data may further improve its clinical relevance.

## 1 INTRODUCTION

Alzheimer’s Disease (AD) may start in the brain years before clear symptoms are seen. In the early stages, imaging findings can also be very subtle [1, 2]. For this reason, a diagnosis made only after obvious clinical symptoms appear may be too late to reflect the earlier course of the disease. Therefore, biomarkers capable of capturing disease progression at earlier stages are critically needed [3]. In clinical settings, disease staging and longitudinal follow-up are rarely based on a single finding. Cognitive assessments are commonly interpreted alongside imaging and biofluid evidence when distinguishing AD from other forms of dementia. Structural Magnetic Resonance Imaging (MRI) plays a central role in this process by providing a non-invasive view of regional brain atrophy patterns that evolve over the course of the disease, while additional biological information may be obtained from molecular imaging techniques and fluid-based markers when such data are available [3-6].

These findings support the need for stage-sensitive modeling strategies, although the present study is intentionally limited to 2D structural MRI slices.

Recent systematic reviews have further emphasized the need for translational computerized clinical decision support systems that integrate imaging-based staging into clinical workflows [5]. Blood-based biomarkers can be collected more easily than PET imaging or CSF measures and can be repeated with less burden for patients. For this reason, they are increasingly considered in clinical and research settings. However, information obtained from different biomarkers does not always indicate the same disease stage. Recent evidence has shown that plasma biomarkers are associated with both cerebral perfusion and structural brain measures in AD [6]. These relationships indicate that biofluid and imaging markers may capture partly distinct but complementary aspects of disease status. Bringing these heterogeneous signals together in a consistent way therefore remains a challenge. Beyond imaging-only pipelines, multi-diagnostic machine learning approaches have also been proposed for early Alzheimer detection with an emphasis on generalizability across settings [7]. Structural MRI has also been used as input to learning-based models to examine whether image characteristics differ systematically between cognitively normal individuals and patients assigned to distinct stages of AD [8, 9]. Several recent studies have reported results using deep Convolutional Neural Network (CNN) backbones and transfer learning for multi-class Alzheimer staging from MRI data [10, 11]. Early-stage prediction has also been explored with classical and deep learning models as a clinically important screening direction [12]. In parallel, transformer-based architectures have been considered in MRI-based studies to assess whether modeling relationships between image slices contributes additional information for stage-level classification [13]. In clinical practice, disease staging does not rely on isolated decisions. Earlier assessments usually influence how later stages are interpreted. When the sequential course of the disease is not taken into account, results across disease stages become harder to interpret consistently. Previous studies have reported good results with transfer learning, ensemble methods, and multi-stage designs. However, most of these methods still treat each diagnostic stage as an independent classification problem. In sequential learning settings, this is still challenging because earlier knowledge can be lost through catastrophic forgetting. In this work, a unified framework is introduced to support hierarchical AD staging in a continual-learning manner, where related tasks are learned progressively while earlier diagnostic knowledge is preserved. In particular, retention is encouraged through regularization together with a compact rehearsal buffer, avoiding large-scale storage or full replay of all prior patient data.

- This study used a nested multi-head ResNet-18 model to learn Alzheimer staging tasks step by step from structural MRI.
- Elastic Weight Consolidation (EWC) is incorporated to reduce catastrophic forgetting when tasks are introduced sequentially.
- Progressive layer unfreezing and focal loss are adopted to improve stability during incremental training and to address class imbalance.

### 1.1 Related Work

Structural MRI has been widely used in recent studies to support automated staging of Alzheimer’s disease, as regional atrophy patterns contain information relevant to diagnosis. Many deep learning studies have used MRI data to learn patterns related to Alzheimer’s disease. A ResNet-based transfer-learning study on a four-class MRI dataset reported an overall accuracy of 98.35% with ResNet101v2 [10]. Explainable machine learning frameworks have also been proposed to improve clinical interpretability in Alzheimer MRI classification [14]. Knowledge distillation has further been investigated as a strategy for transferring diagnostic knowledge into lightweight student models for Alzheimer MRI classification [15]. These studies show that MRI-based patterns can help classify Alzheimer’s disease. However, the results may change depending on the dataset, how the labels are defined, and which preprocessing steps are used [8, 9]. Recent studies have also discussed newer deep learning approaches for improving early Alzheimer diagnosis from MRI data [16]. An ensemble transfer-learning strategy combining features from multiple pretrained CNN backbones achieved an average accuracy of 97.93% on a four-stage Alzheimer MRI classification benchmark [11]. Most studies were evaluated in static settings. In this work, staging was formulated as a nested, sequential set of tasks, and retention was quantified as forgetting.

Beyond conventional convolutional architectures, transformer-based models have been introduced to examine whether relationships between MRI slices contribute additional information for stage-level classification [13]. More complex cascaded transformer architectures have also been proposed for multimodal Alzheimer classification under incomplete data conditions [17]. Class imbalance is a common problem in Alzheimer’s disease datasets. Several studies have sought to reduce this problem by using imbalance-aware model designs or modified loss functions, so that performance remains more stable across classes [18]. Severity grading under imbalanced class distributions has also been investigated using transfer-learning models combined with augmentation strategies [19]. In most previous studies, stage refinements were learned separately. In the present study, finer decisions were learned step by step, while earlier screening performance was kept as stable as possible.

Another group of studies has examined MRI data collected at multiple time points and from different imaging sources. The goal of these works is to reflect better how Alzheimer’s disease changes over time rather than relying on a single snapshot. These approaches aim to capture temporal changes while still allowing each task or modality to contribute distinct information [20, 21]. Related multimodal feature-selection methods have also been proposed to improve robustness by learning cross-modality similarity structure rather than relying on fixed priors [22]. Hypergraph-based formulations, in particular, have been used to encode higher-order relationships among subjects and modalities, with multi-task learning applied to extract shared discriminative patterns for Alzheimer’s disease classification [23]. Instead of temporal acquisition in data, temporal acquisition in tasks was modeled and evaluated. In many MRI-based studies, Alzheimer’s disease stages are predicted in a single step, and each diagnostic category is treated as a separate class. Other studies have used multi-stage designs. In these designs, the model first makes a general screening decision and then performs a more detailed stage-level classification. A cascaded CNN framework has also been reported for progressive Alzheimer staging, with high classification accuracy [24].

Retention across stages was rarely measured. In this study, forgetting was reported under sequential stage acquisition. Clinical diagnosis, however, usually proceeds in a sequential manner, with decisions made at earlier stages influencing how later stages are interpreted. Related multi-stage diagnostic frameworks have also been proposed to refine Alzheimer staging progressively [25]. Hierarchical classification methods have also been studied for Alzheimer diagnosis. Earlier work used handcrafted fusion descriptors within a structured decision hierarchy [26]. More recent work has introduced hierarchical vision transformer models to include severity-aware information in Alzheimer staging [27]. Sequential feature embedding has also been explored for distinguishing prodromal Alzheimer stages [28]. This idea is consistent with broader hierarchical medical imaging studies, where disease categories and severity levels are encoded to improve consistency and interpretability across related classes [29–31]. Several recent review studies have pointed out that results reported for Alzheimer’s disease classification do not always remain consistent when applied to different datasets, especially when label definitions, class balance, and evaluation protocols vary [32].

Finally, continual learning has been discussed as a suitable paradigm for clinical decision-support systems that require periodic updates while preserving prior knowledge. Sequential training in medical image analysis has been shown to cause the loss of previously learned information when new tasks are introduced, a phenomenon commonly referred to as catastrophic forgetting. To address this issue, different strategies have been proposed, including parameter regularization, reusing past data, and architectural separation between tasks [33]. A shared-backbone multi-head design was adopted. Stability was improved using EWC and compact replay.

In privacy-sensitive medical settings, storing or replaying prior patient data is often not feasible. Under these conditions, maintaining performance across tasks has been reported to be difficult, which has led to increased attention on regularization-based methods, such as constraints inspired by Elastic Weight Consolidation, as practical alternatives [34]. Full data replay was not assumed. A compact replay buffer was used to support retention, together with regularization. Studies on class-incremental learning have shown that evaluation results may depend on the training setting and task order. These studies also highlight the difficulty of balancing stability with adaptation to new tasks [35, 36]. These findings indicate that Alzheimer’s disease staging may benefit from learning schemes in which new stages are introduced gradually. In such settings, it becomes important to retain information learned at earlier stages while new distinctions are added. Overall, sequential staging was evaluated, and retention was explicitly quantified.

## 2 MATERIALS AND METHODS

Because AD progresses over time, clinical assessment often follows a stepwise pattern. A broad screening decision is usually followed by more detailed judgments about disease severity. In this study, this idea was reflected in the learning protocol. For this purpose, nested binary tasks were created and trained in a fixed order. The model used a shared backbone with separate task-specific heads. Progressive unfreezing, focal loss, EWC, and compact replay were used to stabilize sequential training.

### 2.1 Problem Formulation And Task Construction

AD stage recognition was formulated as a nested continual learning problem over 2D brain MRI images. Let;


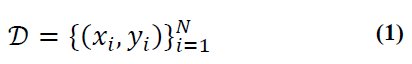


where xᵢ denotes an MRI slice and yᵢ ϵ {0,1,2,3} denotes the clinical label: 0 = Non-Demented, 1 = Very Mild Demented, 2 = Mild Demented, 3 = Moderate Demented [37]. From D, three nested binary tasks {T1, T2, T3} were derived to reflect clinically meaningful refinements (Fig. **1**), and training was carried out sequentially as T1 → T2 → T3.

The tasks were performed from general to specific, starting with a model-screening task and then progressing to more specific distinctions within clinically significant groups. This was done to prevent sudden changes in the learning objective and to help the model retain useful features across relevant subtasks. It also makes it easier to interpret forgetting results, since any drop in performance can be linked to a clinically meaningful refinement rather than to an arbitrary split of the classes. For that reason, T1 -> T2 -> T3 was treated as the clinically motivated primary learning protocol. It should be interpreted as a methodological ordering for nested stage refinement, not as a biological model of Alzheimer disease progression.

Task T1 (Screening): A screening task was defined to separate cognitively normal cases from dementia-related cases:


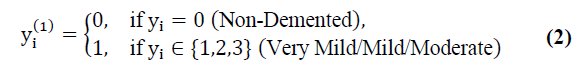


Task T2 (Early-stage discrimination): An early-stage discrimination task was defined on the restricted subset:


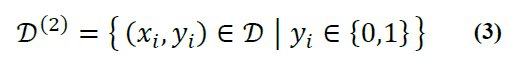


with labels remapped as:


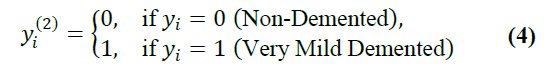


Task T3 (Advanced-stage discrimination): An advanced-stage discrimination task was defined on:


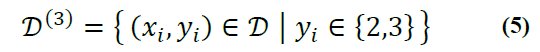


with the remapped target:


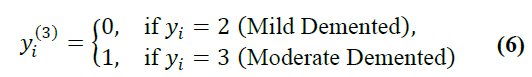


Sequential learning required preserving performance on earlier tasks while optimizing later tasks. Catastrophic forgetting was mitigated through EWC [38] and compact replay-based rehearsal.

### 2.2 Nested Multi-Head Architecture

A shared ResNet-18 backbone was used with a separate classification head for each task. The backbone extracted common image features, while the task-specific heads produced the predictions for T1, T2, and T3. The ResNet-18 encoder was pretrained on ImageNet [39, 40] and was used as the feature extractor fθ(·). Each head (h1, h2, and h3) was implemented as a linear layer on top of the pooled backbone output. For an input x and task index k ϵ {1,2,3}, the logits were computed as:


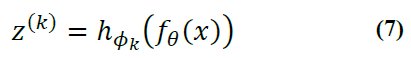


Class probabilities were obtained *via* softmax. The backbone parameters were denoted by θ and head parameters by ϕk. This choice was motivated by the role of the backbone as the shared cross-task representation space. In the multi-head formulation, each binary head is task-specific and relatively small, whereas catastrophic interference is expected to arise primarily from updates to the shared representation. Therefore, Fisher regularization was applied to backbone parameters to reduce representation drift, while task-specific decision boundaries were supported through replay of stored exemplars from previous tasks.

### 2.3 Preprocessing, Augmentation, and Optimization

All images were resized to 224 × 224 pixels and then normalized according to the ImageNet mean and standard deviation values [40]. As MRI slices are single-channel, inputs were converted to three channels to match the ImageNet-pretrained backbone interface. For training, random horizontal flips and random rotations within ±10° were applied. For hold-out testing, the images were only resized and normalized. In cross-validation, random augmentation was added only to the training folds. The validation and test folds were kept the same, with only resizing and normalization so that performance could be evaluated more fairly.

Model optimization was performed with Adam [41]. The Adam hyperparameters were beta1 = 0.9 and beta2 = 0.999. Weight decay was set to 1 x 10^-4, the mini-batch size was 16, the replay mini-batch size was 8, and gradient clipping was set to 5.0. Separate learning rates were assigned to the shared backbone and the task-specific heads, with values of 1 x 10^-4 and 3 x 10^-4, respectively. The learning rate was reduced using ReduceLROnPlateau with a factor of 0.2, patience of 3, and minimum learning rate of 1 x 10^-6. Early stopping monitored validation AUC with patience of 7 and minimum improvement of 5 x 10^-4.

#### 2.3.1 Training Schedule and Fisher Estimation

The proposed model was trained in the fixed order T1 → T2 → T3. Each task was allowed a maximum of 30 epochs. Training could stop earlier when the validation AUC did not improve. The complete hyperparameter and Fisher-estimation settings are provided in Table **S1a** and Algorithm **S1**, while task-wise epoch completion and the progressive-unfreezing schedule are summarized in Table S**1b**.

Fisher estimation was performed after each task and before training started on the next one. The checkpoint from the completed task was used for this calculation. The model remained in evaluation mode, and the training split of the same task was passed through the network without random augmentation. For each mini-batch, cross-entropy loss was taken from the active task-specific head. Backpropagation was used to collect the gradients, and only the squared gradients of the shared backbone parameters were accumulated. The task-specific heads were excluded from this estimate because the EWC penalty was applied only to the shared representation.

Fisher estimation was capped at 2,048 training slices per task or at the full training loader if fewer samples were available. The accumulated squared gradients were normalized by the number of slices used to obtain the diagonal Fisher estimate. The corresponding backbone parameter snapshot and Fisher diagonal were stored before training the next task. During optimization of a new task, only Fisher matrices from previously learned tasks were active; the Fisher matrix of the current task was not used in its own EWC term. The EWC regularization weight was λ = 2,000.

### 2.4 Progressive Unfreezing Schedule

Progressive fine-tuning was used during sequential training. The aim was to avoid changing too many backbone layers at the beginning. For T1, only Layer4 was trained. For T2, Layer 3 and Layer 4 were trained. For T3, training was extended to Layer2–4. Thus, the model first updated the deeper layers and then gradually allowed more of the backbone to adapt in later tasks.

### 2.5 Focal Loss For Imbalanced Subtasks

Focal loss [42] was used as the primary optimization objective for all tasks. Let p ϵ (0,1) denote the predicted probability for the positive class and define:


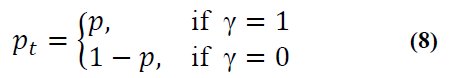


Focal loss was given by:


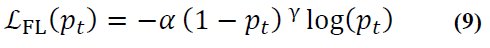


Here, γ = 2 was used to emphasize hard examples *via* the focusing term; α = 1 was fixed for all three tasks. No per-task tuning of γ or α was performed in the primary experiments; class imbalance was addressed primarily through focal modulation, stratified splitting, and class-balanced replay selection.

### 2.6 Elastic Weight Consolidation

Only the shared backbone was regularized with EWC [38]. This was done to limit changes in backbone weights that had been useful for earlier tasks. The diagonal Fisher estimate was calculated with cross-entropy loss, following the original EWC formulation. During task training, focal loss was used instead, because the classes were imbalanced. In this way, cross-entropy was used for Fisher estimation, while focal loss was used for optimization. For task Tt, the objective was written as:


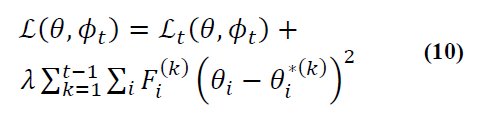


The objective was minimized, where Lt denotes the task loss (focal loss) and λ controls the stability–plasticity trade-off. In the reported configuration, λ = 2000 was used. Importantly, the EWC penalty was applied only using Fishers from previously learned tasks (the current task Fisher was excluded during its own optimization).

The detailed Fisher-estimation procedure is described in Section 2.3.1. In brief, task optimization used focal loss, whereas Fisher estimation used cross-entropy on the deterministic training split to remain consistent with the standard EWC formulation. When training a new task, only Fisher terms from previously learned tasks were active; the current task’s Fisher matrix was stored only after that task had been completed and was therefore excluded from its own EWC penalty.

Additional experiments were performed to check whether EWC had to be limited to the backbone. For this purpose, backbone-only EWC was compared with EWC applied to all trainable parameters, including the task-specific heads. A separate knowledge-distillation setting was also tested. In that setting, LwF-style logit distillation was used with T = 2.0, without EWC or replay. These analyses are reported in Section 3.4.

### 2.7 Replay-based Rehearsal

Replay was combined with EWC to help retain information from earlier tasks. After each task, up to 200 preprocessed samples were kept in the buffer. The stored samples were resized and normalized before storage. No random augmentation was applied at this stage, which kept the replay examples fixed during later training. The buffer size of 200 samples per task was chosen as a practical memory limit. It was used to keep memory cost low while preserving representative examples from earlier tasks. This value was fixed for the main experiments and was not treated as an optimized setting. Replay memory size and sample selection were examined separately to determine their effects on retention. Buffer sizes of 0, 100, and 200 samples per task were compared. For non-zero buffer sizes, class-balanced sampling and herding were also evaluated. When the buffer size was 0, no samples were stored, so all selection strategies were equivalent to the no-replay setting.

The replay-buffer size and selection-strategy sensitivity results are reported in Table **S2**. During training on task Tt, small replay mini-batches (size 8) were drawn from each previously learned task and evaluated using the corresponding historical head. The replay loss used the same focal-loss criterion as the main task loss and was combined with the task loss and EWC penalty:


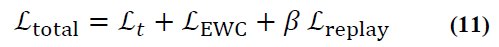


Where denotes a scalar weight. In the reported configuration, replay was enabled by default with β = 0.5.

### 2.8 Evaluation Protocol, Baselines, and Component Ablations

Performance was evaluated on the fixed hold-out split provided by the dataset, using deterministic test preprocessing. Folds were created on the provided training split using stratification on the Level 1 labels. Because subject identifiers are not available in the released dataset, we could not enforce subject-level separation. Therefore, slices from the same subject may appear in both the training and validation/test partitions, potentially inflating performance estimates. All reported results should be interpreted as slice-level generalization, not subject-level clinical generalization. Accuracy and ROC–AUC were reported for each level. After the full T1 -> T2 -> T3 training sequence, all task heads were tested again with the final shared backbone. This evaluation was used to measure how well earlier tasks were retained in terms of discrimination and calibration. Along with accuracy and ROC-AUC, sensitivity, specificity, Brier score, and Expected Calibration Error (ECE) were also reported. The operating threshold for each task was chosen on the validation split using the Youden rule. The same threshold was then used on the test split. ECE was calculated with fixed probability bins, and the Brier score was included as an additional calibration measure. Performance matrices and curves were generated for the proposed method at the checkpoint immediately after training the corresponding level (*i.e*., T1 after Level 1 training, T2 after Level 2 training, and T3 after Level 3 training). Forgetting was quantified as the decrease in accuracy on an earlier task between the time it was learned and the final sequential model after subsequent tasks were introduced.

To assess the individual contributions of EWC and replay, four sequential experimental conditions were examined using the same task order, data splits, epoch budget, early-stopping criterion, and optimization settings: the proposed EWC + replay configuration, EWC-only, replay-only, and no EWC/no replay. In the no EWC/no replay setting, both the EWC penalty and replay rehearsal were removed. This setting corresponds to the previously reported “No EWC” ablation; it is renamed here to make clear that replay was also disabled. In the EWC-only setting, the EWC penalty was kept while replay was omitted. Conversely, in the replay-only setting, rehearsal from the compact replay buffer was retained, whereas the EWC penalty was disabled. Extra ablation analyses were included to examine the scope of EWC and distillation. For EWC scope, backbone-only EWC + replay was compared with all-parameter EWC + replay. For distillation, an LwF-style KD setting was tested with temperature T = 2.0. In this setting, EWC and replay were not used, so the effect of KD could be examined on its own.

The comparison involved several continual-learning approaches: Learning without Forgetting (LwF) with logit-based distillation, Synaptic Intelligence (SI), Memory Aware Synapses (MAS), replay-only experience replay (ER), A-GEM, DER++, and a prototype-based classifier following the iCaRL framework. Except where a different setting is explicitly specified, all approaches were evaluated using identical task definitions and ordering, the same data partitions and preprocessing pipeline, and comparable backbone architectures. These comparisons were used to position the proposed EWC + replay design relative to common continual-learning methods. They were not intended to show state-of-the-art performance across all continual-learning metrics. The accompanying Supplementary Material presents the findings from the additional analyses. EWC-scope and knowledge-distillation ablations are summarized in Table **S3**. Five-fold slice-level cross-validation results are provided in Table **S4**. Detailed continual-learning baseline metrics are reported in Table **S5**, while non-continual, hierarchical joint, architecture, and texture/radiomics baselines are provided in Table **S6**. Task-order sensitivity results are reported in Table **S7**.

### 2.9 External Subject-level Validation

External validation was performed on OASIS-VBM T1-weighted MRI slices because subject identifiers were available in this dataset. The selected OASIS subset included 226 subjects and 1,582 slices. A three-fold cross-validation scheme was used at the subject level, and all slices from the same subject were placed in the same fold. Slice-level probabilities were aggregated for each subject before calculating AUC, sensitivity, specificity, ECE, and Brier score. The OASIS folders did not provide separate mild and moderate dementia groups. For this reason, the OASIS severity task was defined as CDR0.5 versus CDR1+, rather than the exact mild-versus-moderate task used in the Mendeley slice-level analysis. The OASIS subject-level dataset audit and detailed task mapping are provided in Table **S8**. The reproducibility artifacts and audit files associated with the experimental workflow are listed in Table **S9**.

## 3 RESULTS

All Mendeley results in this section are reported as slice-level methodological findings because the released Mendeley dataset does not provide verified subject identifiers. They are therefore not interpreted as patient-level clinical performance. Subject-level generalization was evaluated separately using OASIS-VBM, as reported below:

### 3.1 Slice-level Post-sequence Performance and Component Ablation

After completing the full T1 → T2 → T3 sequence, all task-specific heads were re-evaluated with the final shared backbone. The resulting slice-level ROC curves for the proposed EWC + replay model are shown in Fig. (**2**).

The ROC curves show high slice-level discrimination after the full sequence, with post-sequence AUC values of 0.976, 0.985, and 1.000 for T1, T2, and T3, respectively. These values are reported as methodological slice-level performance and should be considered together with the calibration and subject-level OASIS results below.

The corresponding post-sequence confusion matrices are shown in Fig. (**3**).

The confusion matrices indicate balanced retention for T1 and T2, whereas the T3 matrix should be interpreted cautiously because the advanced-stage subset contains substantially fewer positive samples. Training dynamics across the sequential tasks are shown in Fig. (**4**).

Loss changes were expected at the points where the model moved from one task to the next. At these boundaries, the classification target changed, and the consolidation and replay terms also became active. For this reason, the increase in validation loss near the start of the final task was interpreted as a normal task-transition effect, not as a problem caused by an arbitrary epoch choice. In the main run, training stopped after 30 epochs for T1, 23 epochs for T2, and 12 epochs for T3, following the early-stopping rule described in the Methods section. The corresponding training-stability and loss-spike audit is reported in Table **S10**. Component-level retention is summarized in Table **1**.

The ablation results showed that the earlier “No EWC” setting actually removed both EWC and replay. Replay was the main factor supporting retention in this protocol. EWC alone led to higher forgetting. The combined EWC + replay model remained competitive, but replay-only achieved similar or better results on some forgetting metrics. Post-sequence discrimination and calibration metrics for the proposed model are reported in Table **2**.

In the Mendeley slice-level evaluation, discrimination remained high after the full task sequence. However, calibration varied across tasks. For T3, the higher ECE and lower sensitivity may be related to the smaller and more imbalanced advanced-stage group. These results add useful information beyond accuracy-based forgetting, but they should not be read as evidence of patient-level clinical performance. Calibration curves for the proposed model are shown in Fig. (**5**).

The reliability diagram supports the numerical calibration results: T1 and T2 show more stable calibration than T3, while T3 probabilities are affected by the limited number of positive advanced-stage examples.

### 3.2 External Subject-level OASIS Validation

Because verified subject identifiers are not available in the Mendeley benchmark, external subject-level validation was performed on OASIS-VBM. The proposed model was evaluated using subject-wise three-fold cross-validation, ensuring that all slices from a given subject remained within the same fold. The OASIS AUC summary for the proposed EWC + replay configuration is shown in Fig. (**6**).

OASIS performance was substantially more moderate than the Mendeley slice-level results, which supports the revised interpretation that the Mendeley benchmark is useful for method development but not sufficient for patient-level clinical validation.

Subject-level OASIS results for all component configurations are reported in Table **3**.

The OASIS results did not indicate a single best component setting across all tasks. EWC-only gave the highest T1 AUC, replay-only gave the highest T2 AUC, and the proposed EWC + replay setting achieved the highest T3 AUC. This pattern suggests that the framework should be viewed mainly as a continual-learning pipeline for AD staging. Further subject-level validation would still be needed before drawing clinical conclusions.

### 3.3 Non-continual and Handcrafted Baselines

The proposed model was also compared with non-continual and handcrafted baselines, as shown in Table **4**. This comparison was included to assess whether sequential learning provided an advantage over static training.

The static baselines also gave competitive slice-level AUC results, especially for T3. However, these models were not designed to evaluate how well earlier tasks are retained after new tasks are learned. For this reason, the main contribution of the proposed framework is not simply higher raw performance than every static model. Its value is that it provides a reproducible continual-learning setting in which forgetting can be measured explicitly.

### 3.4 Continual-learning Baseline Comparison

The proposed method was compared with other continual-learning approaches. Figure **7** shows the final accuracy results, and Table **5** lists the final AUC and forgetting values.

Figure **7** shows that LwF and DER++ were strong baselines in this slice-level protocol, whereas A-GEM, MAS, and SI showed lower retention for earlier tasks.

LwF and DER++ gave strong results, and in some forgetting measures they performed better than the proposed setting. For this reason, the proposed method is not presented as the best continual-learning method in every respect. Its main contribution is the nested AD staging setup, the clear ablation design, the compact replay strategy, and the reproducible experimental package.

### 3.5 Task-order sensitivity

The task order was also tested, since the main T1 → T2 → T3 sequence follows the clinical staging logic but may not yield the best result for every slice-level metric. The comparison is given in Table **6**.

The order of tasks affected final slice-level performance, confirming that task order is not a neutral implementation detail. The T1 → T2 → T3 order was retained as the primary protocol because it represents a coarse-to-fine nested decision pathway, not because it was the numerically strongest order or a biological model of AD progression.

## 4 DISCUSSION

In this work, Alzheimer MRI staging was treated as a sequential learning problem rather than as a set of independent classification tasks. The aim was to examine whether information learned in earlier binary tasks could be retained when the model later made finer diagnostic distinctions. The revised experiments show that this setting is useful for studying retention across related AD staging tasks. At the same time, the Mendeley results need careful interpretation, since the dataset was analysed at the slice level and subject-wise independence could not be confirmed.

The ablation results showed a clear role for replay. When replay was used, earlier task performance was better preserved. In contrast, EWC alone led to greater forgetting, indicating that regularization alone was insufficient without rehearsal in this protocol. This result is reasonable, because replay allows the model to revisit representative samples from previous tasks, whereas EWC mainly limits changes in the shared representation. The additional analyses of the EWC scope and knowledge distillation are presented in Table **S3**.

The OASIS results changed the interpretation of the Mendeley findings. When the evaluation was conducted at the subject level, the AUC values were close to 0.75, and calibration errors were still observed. For this reason, the results should be used mainly to judge the method, not to support direct clinical diagnosis. The OASIS results therefore point to the need for further work at the subject level.

Beyond structural MRI, magnetic resonance spectroscopy has been evaluated as a diagnostic biomarker for AD, while DTI-ALPS and hippocampal microstructural measures have been used to characterize stage-specific changes across the AD continuum [43, 44]. These studies provide broader biomarker context, but they do not directly validate the present 2D structural MRI continual-learning framework.

MRI texture-analysis research in AD indicates that quantitative texture features may capture smaller-scale tissue changes beyond conventional volumetric measures [45]. This observation supports future studies combining deep representations with radiomic or texture features, but it does not directly validate the present 2D continual-learning pipeline. Table **7** places the proposed work in the context of recent MRI-based AD studies and references on continual learning.

These results show that 3D CNNs, transformer models, and multimodal approaches should still be explored in future work. In this study, the 2D ResNet-18 model was used mainly to create a controlled continual-learning setting. It was not intended to represent the most advanced architecture for clinical AD diagnosis.

## 5 STUDY LIMITATIONS

The main limitation is that the Mendeley dataset does not include verified subject identifiers. For this reason, subject-wise separation and patient-level forgetting could not be assessed. It was also not possible to fully exclude leakage between splits. The Mendeley results should therefore be interpreted as slice-level findings, and they may be inflated if slices from the same subject appeared in different splits. Although OASIS was added as an external subject-level validation cohort, OASIS did not exactly reproduce the Mendeley label hierarchy. The OASIS severity task was defined as CDR0.5 versus CDR1+ because mild and moderate dementia folders were not separately available. ADNI, longitudinal subject-level cohorts, and full slice-to-volume aggregation remain necessary for stronger clinical validation.

In this study, 2D MRI slices were analyzed with a ResNet-18 backbone. This design was suitable for constructing a controlled continual-learning benchmark; however, it does not cover the broader state of the art represented by 3D CNNs, transformer architectures, or multimodal fusion methods. A 3D CNN baseline was not implemented because reliable subject-wise volume reconstruction could not be performed from the slice-only Mendeley dataset.

The buffer sweep covered the completed configurations of 0, 100, and 200 exemplars per task with class-balanced and herding selection, but not the full grid of 50 and 400 exemplars or all selection strategies at every non-zero buffer size. Fairness and bias analysis could not be performed because demographic variables were not available in the released Mendeley data, and OASIS demographic subgroup analysis was not part of the present revision. These issues should be addressed in future subject-level studies. Further details concerning fairness and uncertainty constraints are provided in Supplementary Note **S1**.

## CONCLUSION

This study introduced a nested continual-learning framework for hierarchical Alzheimer MRI staging, based on a shared ResNet-18 backbone, task-specific heads, EWC, focal loss, progressive unfreezing, and compact replay. The revised analyses showed that replay contributed most to retention. They also showed that calibration, task order, and the difference between slice-level and subject-level evaluation must be considered when interpreting the results. Since OASIS subject-level validation yielded performance that was more moderate than the Mendeley slice-level analysis, the framework is better regarded as a reproducible tool for studying sequential retention in AD staging, rather than a clinically validated diagnostic model.

## Figures and Tables

**Fig. (1):**
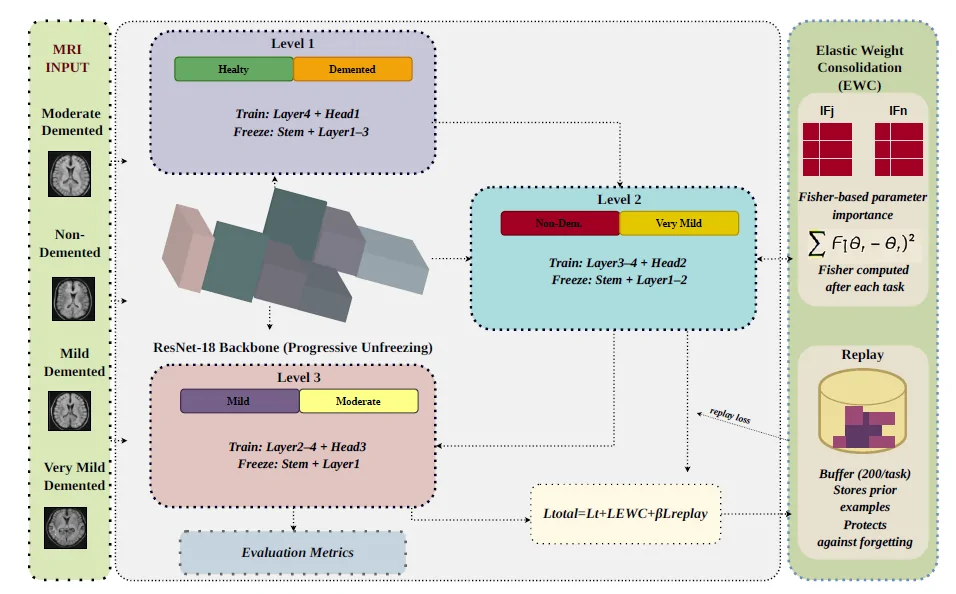
Three-level task decomposition and training order.

**Fig. (2):**
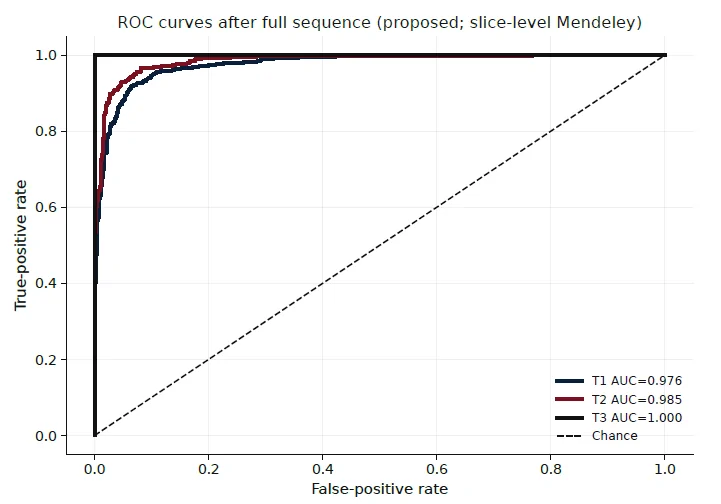
Slice-level ROC curves after the full T1 -> T2 -> T3 sequence for the proposed EWC + replay model on the Mendeley hold-out evaluation. AUC values are shown in the figure legend.

**Fig. (3):**
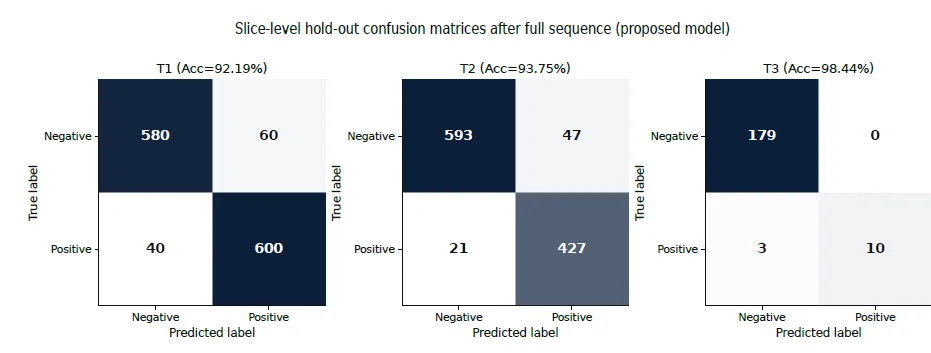
Slice-level hold-out confusion matrices after the full T1 -> T2 -> T3 sequence for the proposed EWC + replay model. Counts are reported separately for T1, T2, and T3 using task-specific decision thresholds.

**Fig. (4):**
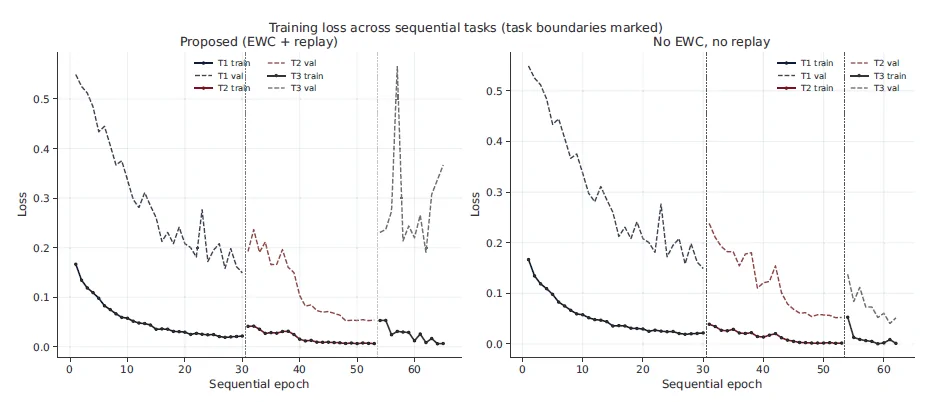
Training loss trajectories for the proposed EWC + replay configuration and the no EWC/no replay ablation. Vertical dashed lines indicate task transitions during the T1 → T2 → T3 sequence.

**Fig. (5):**
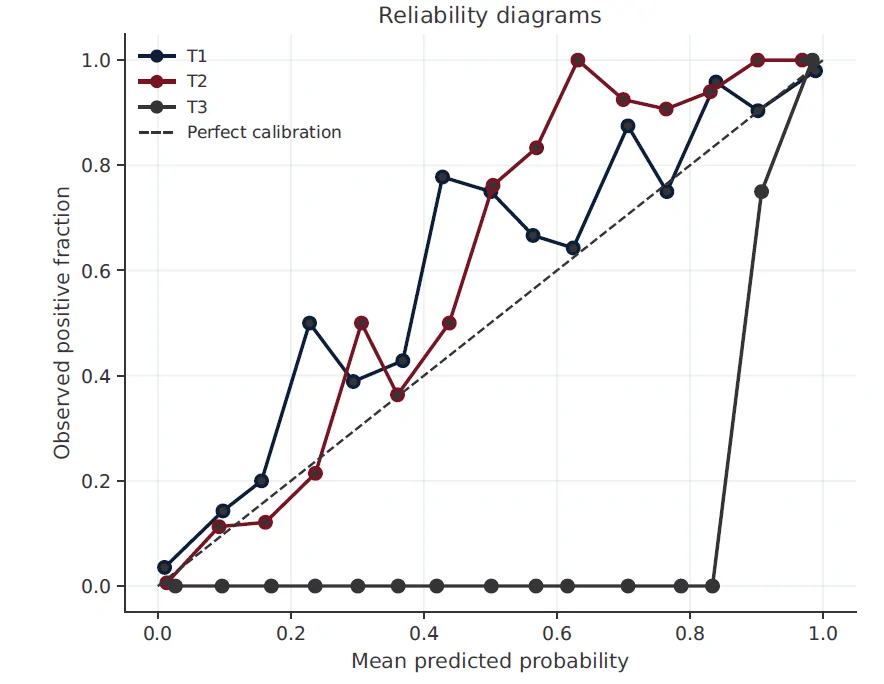
Reliability diagrams for the proposed EWC + replay model after the full sequence on the Mendeley slice-level hold-out split.

**Fig. (6):**
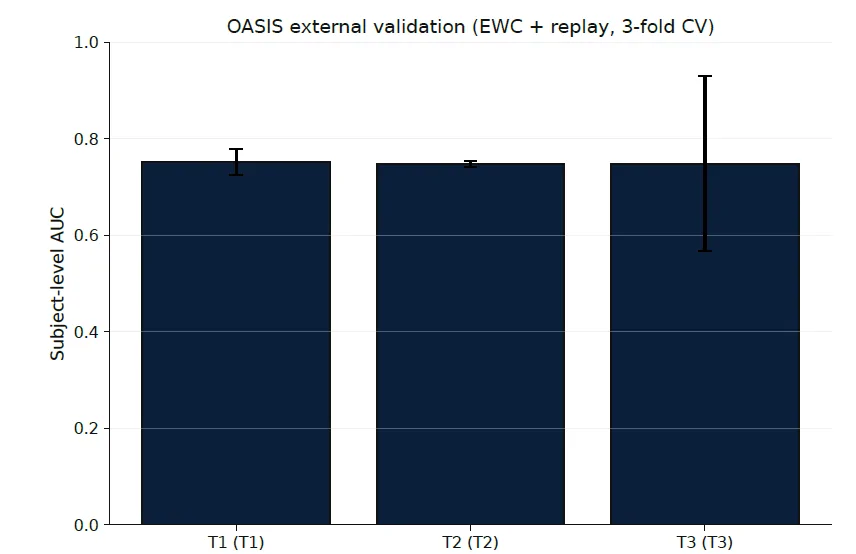
OASIS subject-level external validation for the proposed EWC + replay model using three-fold cross-validation.

**Fig. (7):**
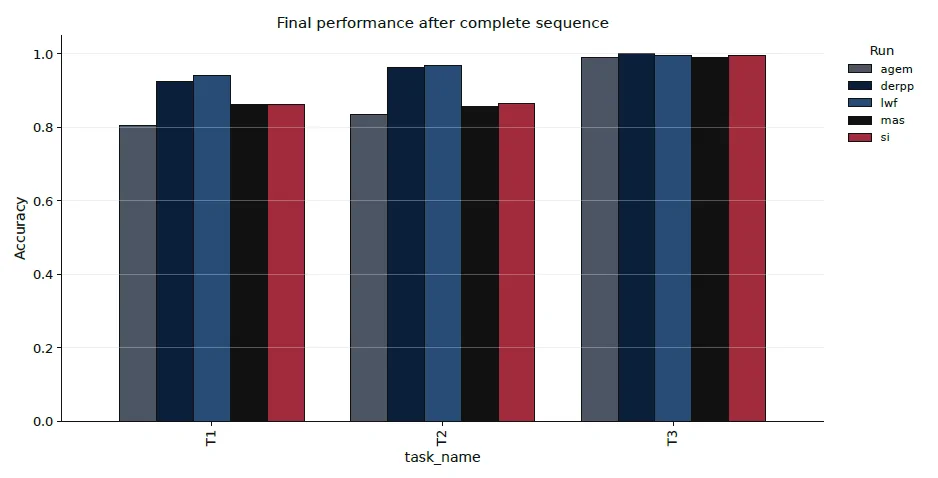
Final slice-level performance after the complete T1 → T2 → T3 sequence for representative continual-learning baselines.

**Table 1:** Component ablation analysis after the full T1 → T2 → T3 sequence. Forgetting is reported as the difference between the accuracy immediately after learning a task and the final accuracy after completing subsequent tasks, in percentage points.

**Method**	**EWC**	**Replay**	**Level 1 forgetting (pp)**	**Level 2 forgetting (pp)**
Proposed EWC + replay	Yes	Yes	4.14	5.24
Replay-only	No	Yes	3.67	4.87
EWC-only	Yes	No	7.42	9.47
No EWC/no replay	No	No	5.31	8.00

**Table 2:** Slice-level Mendeley task-completion and post-sequence performance for the proposed EWC + replay model. Post-sequence metrics were computed after completing the full T1 → T2 → T3 sequence.

**Task level**	**N**	**Acc. at completion (%)**	**AUC at completion**	**Post-seq. Acc. (%)**	**Post-seq. AUC**	**Post-seq. Sens.**	**Post-seq. Spec.**	**ECE**	**Brier**
Binary screening (T1)	1280	96.33	0.995	92.19	0.976	0.938	0.906	0.034	0.061
Early-stage discrimination (T2)	1088	98.99	0.999	93.75	0.985	0.953	0.927	0.057	0.053
Advanced-stage discrimination (T3)	192	98.44	1.000	98.44	1.000	0.769	1.000	0.187	0.079

**Table 3:** External validation performance on the OASIS dataset at the subject level, assessed using three-fold cross-validation with non-overlapping subject groups. AUC values are reported as the mean ± standard deviation across folds, while the remaining subject-level metrics are averaged over the three folds.

**Method**	**Task**	**AUC mean ± SD**	**Acc. (%)**	**Sens.**	**Spec.**	**ECE**	**Brier**
Proposed EWC + replay	T1	0.753 ± 0.027	63.30	0.667	0.610	0.150	0.208
Proposed EWC + replay	T2	0.748 ± 0.007	65.15	0.549	0.710	0.241	0.247
Proposed EWC + replay	T3	0.749 ± 0.182	55.21	0.644	0.514	0.297	0.266
Replay-only	T1	0.745 ± 0.044	64.17	0.615	0.663	0.117	0.212
Replay-only	T2	0.760 ± 0.025	65.66	0.578	0.702	0.244	0.245
Replay-only	T3	0.698 ± 0.067	46.88	0.781	0.338	0.286	0.264
EWC-only	T1	0.782 ± 0.011	64.63	0.646	0.649	0.154	0.211
EWC-only	T2	0.744 ± 0.039	65.66	0.506	0.741	0.194	0.221
EWC-only	T3	0.710 ± 0.147	56.25	0.744	0.482	0.327	0.302
No EWC/no replay	T1	0.778 ± 0.039	66.83	0.698	0.648	0.180	0.217
No EWC/no replay	T2	0.726 ± 0.040	65.15	0.593	0.686	0.260	0.263
No EWC/no replay	T3	0.698 ± 0.125	60.42	0.596	0.599	0.283	0.276

**Note:** For OASIS, T3 corresponds to CDR0.5 versus CDR1+ because the available OASIS folders did not separate mild and moderate dementia.

**Table 4:** Non-continual and handcrafted baselines on the Mendeley slice-level benchmark.

**Method**	**Setting**	**T1 AUC**	**T2 AUC**	**T3 AUC**	**Interpretation**
Proposed EWC + replay	Sequential continual learning	0.976	0.985	1.000	Retention and forgetting explicitly measured
Joint multi-class ResNet-18	Static non-continual	0.957	0.945	1.000	Strong static baseline
Hierarchical joint ResNet-18	Static hierarchical	0.960	0.955	1.000	High AUC but lower T1/T2 sensitivity
Texture/radiomics RF	Handcrafted baseline	0.857	0.845	0.889	Complementary but not superior

**Table 5:** Continual-learning baseline comparison after completing the full T1 → T2 → T3 sequence on the Mendeley slice-level benchmark. Forgetting is reported in percentage points for previously learned tasks.

**Method**	**T1 AUC**	**T2 AUC**	**T3 AUC**	**T1 forget. (pp)**	**T2 forget. (pp)**	**Interpretation**
Proposed EWC + replay	0.976	0.985	1.000	4.14	5.24	Competitive; replay-driven retention
LwF	0.986	0.997	0.992	2.34	1.75	Strong logit-distillation baseline
DER++	0.983	0.994	1.000	3.75	1.84	Strong rehearsal/distillation baseline
SI	0.946	0.953	1.000	10.08	12.41	Higher forgetting
MAS	0.938	0.941	1.000	10.08	12.78	Higher forgetting
A-GEM	0.888	0.920	1.000	15.94	15.53	Weakest retention in this protocol
iCaRL-style prototype	0.842	0.825	0.801	n.r.	n.r.	Prototype-level comparison

**Table 6:** Task-order sensitivity on the Mendeley slice-level benchmark. Metrics were computed after completing each alternative sequence.

**Task order**	**Final T1 Acc. (%)**	**Final T2 Acc. (%)**	**Final T3 Acc. (%)**	**Mean final Acc. (%)**	**Final T1 AUC**	**Final T2 AUC**	**Final T3 AUC**
T1 → T2 → T3	89.69	91.18	99.48	93.45	0.962	0.969	0.999
T3 → T2 → T1	95.23	95.31	99.48	96.68	0.991	0.990	0.999
T2 → T1 → T3	93.36	95.77	97.92	95.68	0.987	0.992	0.994

**Table 7:** Comparison with representative recent MRI-based Alzheimer’s disease studies and continual-learning references.

**Reference**	**Data**	**Setting**	**Reported Performance/ Role**
[11]	MRI, four-stage benchmark	Static multi-class ensemble transfer learning	Average accuracy 97.93%
[24]	MRI	Two-stage cascade; no retention analysis	Accuracy 98.24%/ 99.70%
[27]	MRI (OASIS)	Hierarchical vision transformer; not continual learning	Accuracy 92.4%; F1 91.9%
[13]	MRI (ADNI)	ViT + transformer sequence modelling in static tasks	Evaluated on binary and multi-class tasks
[33]	Medical image analysis	Continual-learning survey	Emphasizes protocol and metric sensitivity
[34]	Medical image classification	Privacy-preserving continual learning	Motivates limited-replay settings
**This work, Mendeley**	**6,400 MRI slices**	**Nested ResNet-18; EWC + replay; slice-level method-development benchmark**	**Post-seq. AUC T1/T2/T3: 0.976/0.985/1.000; forgetting T1/T2: 4.14/5.24 pp**
**This work, OASIS**	**226 subjects, 1,582 slices**	**Subject-wise three-fold external validation**	**AUC T1/T2/T3: 0.753/0.748/0.749 for EWC + replay**

## Data Availability

The Mendeley slice-level method-development data used in the primary experiments are publicly available from Mendeley Data as “Alzheimer MRI Preprocessed Dataset,” Version 1, DOI: 10.17632/3r8hw8wmmk.1 (https://data.mendeley.com/datasets/3r8hw8wmmk/1). The dataset is provided by the repository under its own terms. Because verified subject identifiers are not available in the released dataset, all Mendeley analyses in this manuscript are interpreted as slice-level methodological findings rather than patient-level clinical performance. The OASIS-VBM data used for subject-level external validation were obtained from the Open Access Series of Imaging Studies (OASIS; https://sites.wustl.edu/OASISbrains/) and are available to qualified researchers subject to the applicable OASIS access procedures and Data Use Agreement. No original OASIS imaging files are redistributed with this manuscript. Derived non-identifiable result tables, split/audit summaries, and reproducibility artifacts are described in the Supplementary Material. The implementation code, preprocessing scripts, and run logs generated by the authors can be made available from the corresponding author [A.A.] upon reasonable request, subject to repository licenses, OASIS data-use conditions, and institutional restrictions.

## LIST OF ABBREVIATIONS

Acc.: Accuracy
AD: Alzheimer’s Disease
ADNI: Alzheimer’s Disease Neuroimaging Initiative
A-GEM: Averaged Gradient Episodic Memory
AUC: Area Under the Receiver Operating Characteristic Curve
CDR: Clinical Dementia Rating
CNN: Convolutional Neural Network
CSF: Cerebrospinal Fluid
CV: Cross-Validation
DER++: Dark Experience Replay++
DTI-ALPS: Diffusion Tensor Image Analysis Along the Perivascular Space
ECE: Expected Calibration Error
ER: Experience Replay
EWC: Elastic Weight Consolidation
F1: Harmonic Mean of Precision and Recall
iCaRL: Incremental Classifier and Representation Learning
KD: Knowledge Distillation
LwF: Learning without Forgetting
MAS: Memory Aware Synapses
MRI: Magnetic Resonance Imaging
MRS: Magnetic Resonance Spectroscopy
n.r.: Not Reported
OASIS: Open Access Series of Imaging Studies
PET: Positron Emission Tomography
pp: Percentage Points
RF: Random Forest
ROC: Receiver Operating Characteristic
SD: Standard Deviation
Sens.: Sensitivity
SI: Synaptic Intelligence
Spec.: Specificity
VBM: Voxel-Based Morphometry
ViT: Vision Transformer

## SUPPLEMENTARY MATERIAL

Supplementary material is available on the Publisher’s website. These items provide the complete training settings, Fisher-estimation procedure, taskwise epoch completion and progressive-unfreezing schedule, replay-buffer sensitivity, EWC-scope and knowledgedistillation ablations, cross-validation summaries, continuallearning and non-continual baseline comparisons, task-order analyses, OASIS dataset audit and task mapping, reproducibility artifacts, training-stability checks, and fairness and uncertainty constraints.
