# Supplementary material for: Nested Continual Learning with Elastic Weight Consolidation for Progressive Alzheimer’s Disease Classification from MRI
Source: Curr Med Imaging. 2026 Aug 31;22:E15734056512153. doi: 10.2174/0115734056512153260820074645 (PMC13741128; doi:10.2174/0115734056512153260820074645)
Supplement: Supplementary file 1 [file CMIM-22-E15734056512153_SD1.pdf]

1875-6603/26 *Send Orders for Reprints to* *reprints@benthamscience.net*

BENTHAM SCIENCE

CrossMark

Current Medical Imaging

Content list available at: https://benthamscience.com/journals/cmir

Supplementary Material

Nested Continual Learning with Elastic Weight Consolidation for Progressive Alzheimer's Disease Classification from MRI

Abdurrahim Akgündoğdu[1,*] and Şerife Çelikbaş[2]

[1]*Department of Electrical and Electronics Engineering, Istanbul University-Cerrahpaşa, İstanbul, Türkiye*
[2]*Biomedical Device Technology, Namık Kemal University, Tekirdağ, Türkiye*

This supplementary file is intended for publication as supportive/supplementary material. Mendeley results are slice-level unless otherwise stated. OASIS results are subject-level.

Table 1a. Hyperparameters, training schedule, and Fisher-estimation settings.

Category	Parameter	Value
Input	Image size	224 x 224 pixels
Input	Channel handling	Single-channel MRI slices converted to three channels
Normalization	Statistics	ImageNet mean and standard deviation
Augmentation	Training	Random horizontal flip; random rotation +/-10 degrees
Augmentation	Validation/test	Resize and normalization only
Optimizer	Adam beta values	beta1 = 0.9, beta2 = 0.999
Optimizer	Backbone LR	1 x 10^-4
Optimizer	Task-head LR	3 x 10^-4
Optimizer	Weight decay	1 x 10^-4
Optimization stability	Gradient clipping	5.0
Batching	Main mini-batch	16
Batching	Replay mini-batch	8
Scheduler	Type	ReduceLROnPlateau
Scheduler	Factor / patience / minimum LR	0.2 / 3 / 1 x 10^-6
Early stopping	Monitor	Validation AUC
Early stopping	Patience / minimum improvement	7 / 5 x 10^-4
Focal loss	gamma / alpha	2.0 / 1.0; fixed across tasks
EWC	lambda	2,000
Fisher estimation	Loss / split	Cross-entropy on the training split of the completed task
Fisher estimation	Augmentation / scope / maximum samples	No augmentation / backbone only / 2,048 slices per task
Replay	Buffer size / selection / beta	200 samples per task / class-balanced / beta = 0.5
Seed	Random seed	42
Runtime	Execution mode	CUDA if available, CPU fallback; exact environment recorded in training_audit.json

DOI: 10.2174/0115734056512153260820074645, 2026, *22*, e15734056512153

Table S1b. Task-wise epoch completion and progressive unfreezing schedule.

Task	Classification level	Max epochs	Completed epochs	Early stopped	Trainable blocks	Best validation AUC
T1	Healthy vs Demented	30	30	No	layer4	0.992
T2	Non-Demented vs Very Mild Demented	30	23	Yes	layer3 + layer4	0.999
T3	Mild Demented vs Moderate Demented	30	12	Yes	layer2 + layer3 + layer4	1.000

Algorithm S1. Diagonal fisher estimation for backbone-only elastic weight consolidation.

**Input: completed task T_k; training split D_k^train; model checkpoint after task T_k with shared backbone parameters theta_k* and task-specific head parameters phi_k*; active task head h_k; maximum Fisher sample count M = 2048.**
**Output: diagonal Fisher estimate F_k for the shared backbone and the corresponding backbone parameter snapshot theta_k*.**
**Procedure:**
1. Load the checkpoint obtained immediately after completing task T_k.
2. Set the model to evaluation mode and disable stochastic data augmentation.
3. Initialize the Fisher accumulator F_k[j] = 0 for every shared backbone parameter theta_j and set the processed-sample counter n = 0.
4. For each mini-batch (x_b, y_b) from D_k^train while n < M:
4.1. Use at most the remaining M - n samples from the mini-batch.
4.2. Compute logits z_b = h_k(f_theta(x_b); phi_k*) using the active task head.
4.3. Compute cross-entropy loss CE(z_b, y_b) for Fisher estimation.
4.4. Clear previous gradients and backpropagate the cross-entropy loss.
4.5. For each shared backbone parameter theta_j, accumulate the squared gradient: F_k[j] <- F_k[j] + |x_b| (dL/dtheta_j)^2.
4.6. Do not accumulate gradients for task-specific head parameters.
4.7. Update n <- n + |x_b|.
5. Normalize the accumulated squared gradients by the number of processed slices: F_k[j] <- F_k[j] / n.
6. Store F_k together with the backbone parameter snapshot theta_k*.
7. Use the stored pair (F_k, theta_k*) only when optimizing subsequent tasks. The Fisher estimate of the current task is not included in its own EWC penalty.
**EWC penalty in subsequent tasks: L_EWC = (lambda / 2) sum_{r<k} sum_j F_r[j] (theta_j - theta_{r,j}*)^2, with lambda = 2000.**

Note: Fisher estimation used the training split of the completed task, no augmentation, cross-entropy loss, and backbone-only scope.

Table S2. Replay-buffer size and selection-strategy sensitivity.

Buffer size per task	Selection strategy	T1 AUC post-seq.	T1 forgetting (pp)	T2 forgetting (pp)	Interpretation
0	No replay / N.A.	0.965	7.42	9.47	No exemplars stored; all selection rules collapse to no replay
100	Class-balanced	0.978	-1.17	1.93	Best T1 post-sequence AUC in the completed sweep
200	Class-balanced	0.962	0.78	4.96	Fixed 200-sample budget within the sensitivity analysis
200	Herding	0.973	-0.55	3.77	Comparable retention to class-balanced selection at the same capacity

N.A., not applicable. Negative forgetting indicates that the final accuracy after the full sequence exceeded the accuracy immediately after the task was first learned. The final summary did not include the reviewer-suggested 50 and 400 exemplar settings or a full non-zero reservoir/clustering grid; this is acknowledged in the limitations of the manuscript.

Table S3. EWC-scope and knowledge-distillation ablations.

Condition	Scope / KD	Replay	KD	T1 AUC	T2 AUC	T3 AUC	T1 forget. (pp)	T2 forget. (pp)
Backbone-only EWC + replay	Backbone EWC	Yes	No	0.967	0.984	0.999	-2.34	0.28
All-parameter EWC + replay	Backbone + heads EWC	Yes	No	0.970	0.982	1.000	-2.19	1.47
KD only (LwF-style)	None	No	Yes, T = 2.0	0.919	0.944	0.999	5.00	6.34

All-parameter EWC includes head parameters. A separate head-only EWC condition was defined in the ablation plan but was not included in the final reported revision bundle.

Table S4. Five-fold cross-validation summary on the Mendeley slice-level benchmark.

Task level	Mean Acc. (%) +/- SD	Min (%)	Max (%)	95% CI
Binary screening (Healthy vs Demented)	86.74 +/- 1.01	85.35	87.79	[85.85, 87.63]
Early-stage discrimination (Non vs Very Mild)	97.56 +/- 1.46	96.20	100.00	[96.28, 98.83]
Advanced-stage discrimination (Mild vs Moderate)	91.53 +/- 1.96	89.84	94.60	[89.82, 93.25]

This table is reported as a slice-level stability analysis only because verified subject identifiers were unavailable in the Mendeley benchmark.

Table S5. Detailed continual-learning baseline metrics after the full T1 -> T2 -> T3 sequence.

Method	T1 AUC	T2 AUC	T3 AUC	T1 forgetting (pp)	T2 forgetting (pp)
Proposed EWC + replay	0.976	0.985	1.000	4.14	5.24
LwF	0.986	0.997	0.992	2.34	1.75
DER++	0.983	0.994	1.000	3.75	1.84
SI	0.946	0.953	1.000	10.08	12.41
MAS	0.938	0.941	1.000	10.08	12.78
A-GEM	0.888	0.920	1.000	15.94	15.53
iCaRL-style prototype	0.842	0.825	0.801	n.r.	n.r.

The iCaRL-style row is a nearest-mean feature-prototype comparison and is not a full class-incremental iCaRL reproduction; forgetting was therefore not reported for that post-hoc prototype analysis.

Table S6. Non-continual, hierarchical joint, architecture, and texture/radiomics baselines.

Method	Setting	T1 AUC	T2 AUC	T3 AUC	T1 Sens.	T2 Sens.	T3 Sens.	Interpretation
Proposed EWC + replay	Sequential continual learning	0.976	0.985	1.000	0.938	0.953	0.769	Retention and forgetting explicitly measured
Joint multi-class ResNet-18	Static non-continual	0.957	0.945	1.000	0.908	0.864	0.846	Strong static baseline
Hierarchical joint ResNet-18	Static hierarchical	0.960	0.955	1.000	0.348	0.342	1.000	High AUC but low T1/T2 sensitivity
Texture/radiomics random forest	Handcrafted baseline	0.857	0.845	0.889	0.794	0.650	0.154	Complementary but not superior

Table S7. Task-order sensitivity on the Mendeley slice-level benchmark.

Task order	Final T1 Acc. (%)	Final T2 Acc. (%)	Final T3 Acc. (%)	Mean final Acc. (%)	Final T1 AUC	Final T2 AUC	Final T3 AUC
T1 -> T2 -> T3	89.69	91.18	99.48	93.45	0.962	0.969	0.999
T3 -> T2 -> T1	95.23	95.31	99.48	96.68	0.991	0.990	0.999
T2 -> T1 -> T3	93.36	95.77	97.92	95.68	0.987	0.992	0.994

Task order changed final slice-level performance. The primary T1 -> T2 -> T3 order should be read as a clinically motivated nested decision protocol, not as a biological progression model.

Table S8. OASIS subject-level dataset audit and task mapping.

Item	Value
Total subjects	226
Total slices	1,582
NonDemented_CDR0	130 subjects / 910 slices
VeryMildDemented_CDR0.5	68 subjects / 476 slices
MildModerateDemented_CDR1+	28 subjects / 196 slices
T1 mapping	CDR0 vs CDR0.5/CDR1+
T2 mapping	CDR0 vs CDR0.5
T3 mapping	CDR0.5 vs CDR1+ because the available OASIS folders do not separate mild and moderate dementia
Validation protocol	Subject-wise 3-fold cross-validation

Table S9. Reproducibility artifacts and audit files.

Artifact	Purpose
training_audit.json	Machine-readable record of optimizer, scheduler, epoch completion, EWC, replay, and runtime environment
training_T1.csv, training_T2.csv, training_T3.csv	Per-task epoch logs for loss, AUC, learning rate, and threshold selection
final_metrics_after_T3.csv	Post-sequence Mendeley slice-level metrics including AUC, sensitivity, specificity, ECE, and Brier
forgetting_table.csv	Accuracy-based forgetting for continual-learning baselines
buffer_sensitivity.csv	Replay-buffer size and selection-strategy sensitivity
head_consolidation_kd.csv	Backbone-only vs all-parameter EWC and KD isolation
order_sensitivity.csv	Alternative task-order results
noncontinual_baselines.csv	Joint multi-class and hierarchical joint ResNet-18 baselines
texture_radiomics_baseline.csv	Handcrafted texture/radiomics random-forest baseline
summary_by_method_task.csv	OASIS subject-level 3-fold cross-validation summary
subject_level_report.json	Documents that subject-level grouping was not possible for Mendeley due to missing verified subject IDs

Table S10. Training stability and loss-spike audit.

Item	Summary
Primary proposed EWC + replay	No positive epoch-25 instability flagged in the audit; the only flagged entry was an early T1 epoch-2 loss decrease (delta = -0.032, z = -3.918).
No EWC/no replay ablation	Same early T1 epoch-2 loss decrease was flagged; no late positive spike was identified in the reported audit.
Manuscript action	The previous ambiguous loss figure was replaced with task-boundary-marked trajectories, and epoch completion is now reported as T1 = 30, T2 = 23, T3 = 12.

Supplementary Note S1. Fairness and uncertainty constraints.

Fairness and bias analyses could not be performed because the available Mendeley and OASIS revision tables did not include demographic attributes such as sex, age, ethnicity, scanner/vendor, or acquisition-site variables in a form suitable for subgroup analysis. The manuscript therefore does not claim demographic fairness. Calibration was reported using ECE and Brier score, but a full uncertainty-quantification analysis was not implemented and is listed as future work.

© 2026 The Author(s). Published by Bentham Science Publisher.

This is an open access article distributed under the terms of the Creative Commons Attribution 4.0 International Public License (CC-BY 4.0), a copy of which is available at: https://creativecommons.org/licenses/by/4.0/legalcode. This license permits unrestricted use, distribution, and reproduction in any medium, provided the original author and source are credited.

HOW TO CITE:
Akgündoğdu A, Çelikbaş Ş. Nested Continual Learning with Elastic Weight Consolidation for Progressive Alzheimer's Disease Classification from MRI. Curr Med Imaging, 2026; 22: e15734056512153.
http://dx.doi.org/10.2174/0115734056512153260820074645
